# Supplementary material for: Efficient simulation of Time-Fractional Korteweg-de Vries equation via conformable-Caputo non-Polynomial spline method
Source: PLoS One. 2024 Jun 26;19(6):e0303760. doi: 10.1371/journal.pone.0303760 (PMC11207126; doi:10.1371/journal.pone.0303760)
Supplement: Attachment — Submitted filename: Response to Referee.pdf [file pone.0303760.s001.pdf]

Response to Referees

Dear respected referees,

We sincerely thank you for your invaluable comments and feedback on our manuscript. Your expertise and insights have greatly contributed to enhancing the quality of our work. We have carefully considered each of your suggestions and remarks, and we are pleased to inform you that they have been incorporated into the revised version of the manuscript.

Referee 1:

This paper is concerned with the conformable-Caputo fractional non-polynomial spline method for solving the time-fractional Korteweg-de Vries equation. Von Neumann approach demonstrates unconditional stability of the scheme, and its validity is shown. Moreover, quantitative assessment using L_2 and L_∞ error norms confirms its superiority, with worked examples.

The presented results are in current area of research and their appearance in this platform would serve good to the scientific community. In conclusion, this paper is acceptable to appear in Plos One, provided that the subsequent modifications are accounted for in a possible revision:

Comment 1: The language is good overall, requiring some fix in typos and grammars.
Response 1: Thank you for your comment, we checked all.

Comment 2: Introduction could be more informative in terms of novelty and findings.
Response 2: Thank you for your comment, we add a paragraph in the last of introduction to show the novelty and findings .

Comment 3: Differentiate the current work from the recent published (spline methods).
Response 3: Thank you for your comment. The method differs from spline methods in several aspects:

- The construct is based on the non-polynomial spline.
- The conditions contain Caputo fractional derivatives.
- The application is based on conformable derivatives.

Comment 4: The model in (1-3) should be referenced properly from the literature.
Response 4: Thank you for your comment, we added the paragraph to improve the literature of model (1-3) .

Comment 5: The Caputo derivative definition should be given. Why do you call the method Conformable-Caputo? Caputo defition is given in terms of integrals, but Eq.(22) is not!
Response 5: Thank you for your insightful comment, we added the definition of Caputo fractional derivative as Equation (4), about conformable-Caputo derivative, in conditions we used the Caputo derivative and the conformable in application of scheme.

Comment 6: The literature seems weak and not complete missing the latest publications, such as:
https://doi.org/10.1016/j.jocs.2022.101841,
https://doi.org/10.1108/HFF-04-2022-0262,
https://doi.org/10.1016/j.chaos.2022.112980,
DOI: 10.32604/cmes.2022.020781.
Response 6: Thank you for providing the references to recent works that utilize mathematical physics methods to find solutions to the fractional differential equations. We acknowledge the significance of these contributions and have cited two of them in our paper under Ref. [8-9].

Comment 7: Why is the developed method rendering unconditionally stable?
Response 7: Thank you for your insightful comment, because the developed scheme satisfied the Von Neumann stability condition in Equation (37) leads to Equation (38).

Comment 8: Examples chosen are simple and having exact solutions, without dependance on alpha derivative order. Test your method on a more complex KdV model without analytical solution.
Response 8: Thank you for your insightful comment, we chose examples with exact solutions to showcase the effectiveness and suitability of our numerical scheme. In tables and figures, we varied the fractional order alpha to explore the scheme's behavior under different conditions. Moving forward, we'll test our method on more complex KdV models without analytical solutions for further validation.

Comment 9: The Conclusion should be trimmed with focus on the novelty and innovativeness.
Response 9: Thank you for your insightful comment, we revised conclusion according to the comment requirement.

Comment 10: Comparisons with other methods of the conformable-Caputo fractional non-polynomial spline method is not fulfilled.
Response 10: Thank you for your insightful comment, the present work compared with finite difference/collocation method (FDCM) [36] (see Table 1.)

Referee 2:

Before the Editor makes a decision, I suggest that the authors must take into account the following corrections:

Comment 1: I don't think the abbreviations used in the title are inspired.
Response 1: Thank you for your comment, we edited the title to "Efficient Simulation of Time-Fractional Korteweg-de Vries Equation via Conformable-Caputo Non-Polynomial Spline Method".

Comment 2: The "Introduction" section should be more concise.
Response 2: Thank you for your comment, We've condensed the "Introduction" section for improved conciseness.

Comment 3: It is not usual for mathematical relations to appear in the Introduction section.
Response 3: Thank you for your comment, in numerical analysis, it's not uncommon to include mathematical relations in the Introduction section, particularly when introducing the mathematical model that forms the basis of the study. This helps provide context and clarity regarding the problem being addressed and the approach taken to solve it.

Comment 4: Origin of equation (1) is not specified.
Response 4: Thank you for your comment, we added the origin of Equation (1.1).

Comment 5: What is the motivation of the relations (4)?
Response 5: Thank you for your insightful comment, the relations (4) is new non-polynomial spline construct with fractional derivative conditions to investigate the developed numerical scheme.

Comment 6: The expressions for d_j and e_j in (9) and (10) are "bushy". It is difficult to check their correctness.
Response 6: Thank you for your comment, we checked all of them and satisfies Equations (5) and (6).

Comment 7: Details on obtaining relation (19) are required.
Response 7: Thank you for your insightful comment, upon collecting the derivative coefficients of Equation (18) and using Taylor expansion, we get Equation (19)

Comment 8: It is not clear how were obtained the data in Tables.
Response 8: Thank you for your insightful comment, we used L_2 and L_∞ according to Equations (39) and (40).

Comment 9: From where were taken the data used in the graphic representations?
Response 9: Thank you for your insightful comment, we used Software MATLAB and mentioned that in "Numerical illustration and discussion" section

Comment 10: Some editing ”glitches” need to be corrected.
Response 10: Thank you for your insightful comment, we revised paper according to your valuable comment.

Comment 11: References are not uniformly written. In some references the name of the journal is written in full and in other it is abbreviated.
Response 11: Thank you for your insightful comment,

Comment 12: Also, I think, the authors must strengthen the References section with some articles that use some similar techniques, to make the techniques used more plausible, for instance:
On mixed problem in thermos-elasticity of type III for Cosserat media, Journal of Taibah University for Science, 2022; 16(1): 1264–1274;
The Effects of Fractional Time Derivatives in Porothermoelastic Materials Using Finite Element Method, Mathematics. 2021; 9(14): Art. No. 1606;
An evolutionary equation in thermoelasticity of dipolar bodies, Journal of Mathematical Physics, 1999; 40(3): 1391-1399.
Response 12: Thank you for your insightful comment, Thank you for providing the references to recent works that utilize mathematical physics methods to find solutions to the fractional differential equations. We acknowledge the significance of these contributions and have cited two of them in our paper under Ref. [10].

If the authors take into account all these corrections, then this manuscript deserves to be published.

Finally, we express our special thanks to the referees for their helpful comments.

Thank you and best wishes,

Majeed A. Yousif (corresponding author)
